# A novel fast detection algorithm for depression based on 3-channel EEG signals

**DOI:** 10.3389/fnins.2025.1651762

**Published:** 2025-09-29

**Authors:** XiWu Guo, ZiHan Guo, TaoLi Xie

**Affiliations:** ^1^Department of the People's Hospital of Taihe County, Fuyang, Anhui, China; ^2^Medical College, Tianshi College, Tianjin, China

**Keywords:** medically unexplained symptoms, EEG signals, depression, LightGBM, VMD

## Abstract

Medically unexplained symptoms (MUS) are an emerging field in current research. Among middle-aged and elderly patients, most MUS symptoms are mainly caused by depression, but early symptoms do not meet the international somatization standards, which delays treatment. Therefore, developing a rapid auxiliary diagnosis method is of great significance. This paper proposes a novel model for identifying depression based on 3-channel electroencephalogram (EEG) signals from the prefrontal lobe of the human brain. For the collected resting-state EEG signals, variational mode decomposition (VMD) is first used for signal decomposition, and the power spectrum is employed to select intrinsic mode function (IMF) components. After extracting energy features via sample entropy, LightGBM is adopted for classification, with a classification accuracy of 97.42%. Through comparative experiments, the model proposed in this paper achieves a balance between high accuracy and timeliness. This is conducive to the development of a depression detection system based on portable real-time electroencephalography (EEG), and provides a solution for EEG signal devices in real-time depression detection and pre-triage of patients with Medically Unexplained Symptoms (MUS).

## 1 Introduction

The general undifferentiated symptoms refer to pain, fatigue, gastrointestinal and cardiovascular symptoms, which are known as medically unexplained symptoms (MUS), which are very common in elderly people and healthcare patients (Leiknes et al., 2007; Kurita et al., 2012; Steinbrecher et al., 2011; Claassen-van Dessel et al., 2018). These symptoms are generally harmless to the human body, but in recent years, many studies have shown that mental illnesses such as depression often present with medically unexplained symptoms, which may affect their treatment outcomes (Hung et al., 2019; Huijbregts et al., 2010; Simon et al., 1999; Mergl et al., 2007; Barsky et al., 2005; Harris et al., 2009).

Somatic symptom disorder is one of the common mental disorders, with an incidence of ~6% in the general population, particularly among retired or widowed elderly people (Wittchen et al., 2011). According to the diagnostic definition of somatic disorders in the International Classification of Diseases, 10th Edition (ICD-10) (DiSantostefano, 2009), at least six medically unexplained somatic symptoms in two different organ systems, persisting for more than 2 years, are required for a diagnosis of somatic disorder. However, the prevalence of somatic disorder is not high, accounting for only 0.4% in the general population (Creed and Barsky, 2004; de Waal et al., 2004; Fink et al., 2004). Due to the low incidence, many patients with medically unexplained symptoms are often overlooked by hospitals. Meanwhile, studies have shown that many patients with medically unexplained symptoms have some degree of physical impairment but do not meet the strict criteria for somatic symptom disorder, thus failing to receive appropriate treatment (Mayou et al., 2005; Gureje and Reed, 2016). Additionally, regarding fatigue, edema, and unexplained pain as a single condition poses challenges for many professional physicians, mainly because doctors cannot make quantitative judgments based on descriptions (Leiknes et al., 2006; McFarlane et al., 2008; Sharpe et al., 2006), leading to significant differences in diagnostic opinions among physicians.

**Figure 1:**
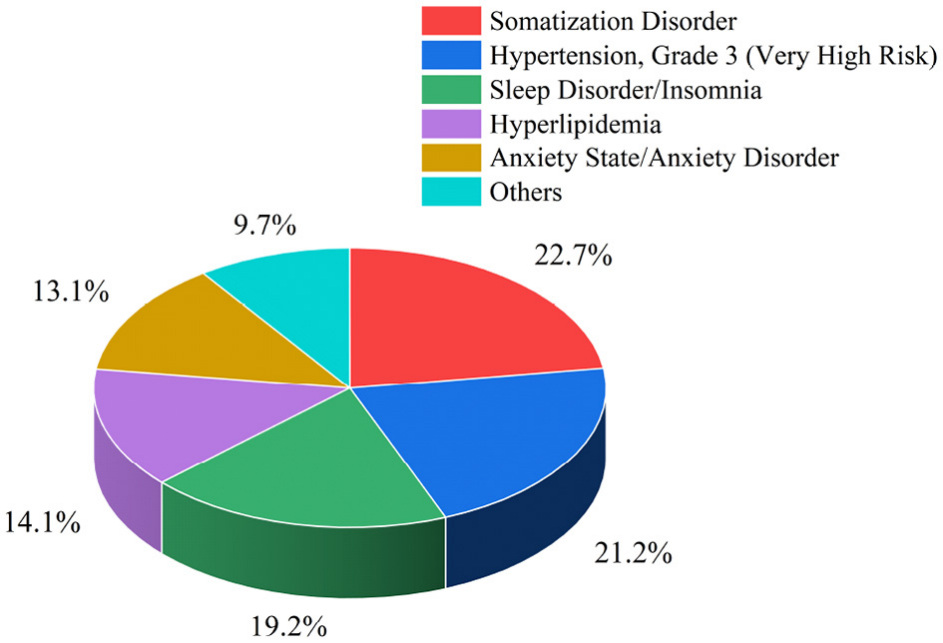
Distribution map of diagnostic results in 198 MUS patients.

Among medically unexplained symptoms, most patients have mental illnesses such as depression. In patients with mild depression, who are in a long-term state of low mood, symptoms are relatively mild and show a certain degree of somatization. These patients have no obvious symptoms and are classified as having medically unexplained symptoms. However, due to the inability to confirm the etiology, patients may fail to receive proper treatment, potentially developing into severe depression and even suicidal ideation. Based on the statistical analysis of medical records of elderly patients admitted to our hospital in the past 5 years, as shown in Figure 1, among a total of 198 middle-aged and elderly patients with medically unexplained symptoms (MUS) admitted, this patient group initially presented with MUS symptoms such as fatigue. Through long-term disease follow-up, the final medically confirmed results showed that 22.7% of the patients had somatization disorder, 21.2% had grade 3 hypertension, 19.2% had sleep disorder of a certain degree, 14.1% had hyperlipidemia, 13.1% had anxiety and anxiety disorder. From the data analysis, nearly 35.8% of the patients initially presented with symptoms such as fatigue of unknown cause, which is likely to be an early manifestation of somatization disorder caused by anxiety and depression. However, according to the quantitative criteria for depression, these patients did not fall into the category of depression in the early stage, which may easily delay treatment. Currently, the most conventional detection method for depression is psychological questionnaires, but it has subjectivity, and due to patients' potential concealment and resistance to psychological questionnaires, accurate diagnostic results are often difficult to obtain (Wang et al., 2023). Therefore, in recent years, electroencephalogram (EEG) signals, as an auxiliary diagnostic tool for mental illnesses, have become a research hotspot.

In recent years, significant advancements in neuroimaging techniques, such as positron emission tomography (PET), magnetic resonance imaging (MRI), and electroencephalography (EEG), have enabled noninvasive studies of brain functions and related disorders (De la Salle et al., 2016). However, the cost of PET and MRI equipment is prohibitive, requiring specialized personnel for operation (Ehman et al., 2017). PET involves the use of radioactive tracers, which increases safety risks and costs (Zhang et al., 2024). Moreover, due to the excessively high costs of PET and MRI, as well as the requirement for professional personnel to conduct interpretation, EEG has become a common technical method for depression detection owing to its advantages such as non-invasiveness and ease of operation (Klooster et al., 2023). The EEG activities in the δ, θ, α, and β frequency bands of patients with depression are usually higher than those in the normal control group, and the α and β frequency bands contain more depression-related EEG information than the low-frequency δ and θ bands (Hasanzadeh et al., 2020). Many scholars have also studied the effects of drugs, environment, religious beliefs, etc., on the brain waves of depressed patients (Berger et al., 2021; Akbari et al., 2021; Zuchowicz et al., 2019; Cao et al., 2019; Grieve et al., 2019; Panier et al., 2020; Feldmann et al., 2018; Bachmann et al., 2018). Mohammadi and Moradi (2021) found through detecting four EEG bands that the Alpha band is closely related to the severity of depression. Nusslock et al. (2018) demonstrated the influence of prefrontal EEG asymmetry on the EEG diagnosis of depression. Zhu et al. (2019) achieved multimodal depression diagnosis by combining EEG and eye movement. These studies focus on the selection of recording locations and EEG frequency bands, and many researchers have also made corresponding contributions in feature extraction.

The identification of depression using EEG signals mainly consists of two aspects: feature extraction and classification models. In terms of feature extraction, it is mainly divided into time-domain and frequency-domain methods. Time-domain methods mainly include techniques such as multiscale principal component analysis, intrinsic time-scale decomposition, linear discriminant analysis, and adjacent component analysis, which are used to analyze EEG time series and extract time-frequency features (Malviya and Mal, 2023). In terms of the frequency domain, Zhang et al. proposed a model combining Wavelet Packet Decomposition (WPD) and Variational Mode Decomposition (VMD) for the extraction of frequency-domain features from EEG signals (Zhang et al., 2024). Alhalaseh and Alasasfeh (2020) applied empirical mode decomposition (EMD) and VMD filters to clean EEG signals and further classified the emotions from EEG signals using entropy and Higuchi's fractal dimension as features. In terms of model classification, many scholars have also made contributions. El-Dahshan et al. (2024) utilized recurrence plots to obtain deep features from PPV signals and demonstrated that recurrence plots can effectively identify periodicity in signals. Siuly et al. (2024) employed the wavelet scattering transform (WST) method to extract time-frequency features of EEG signals, demonstrating the superiority of time-frequency domain features in EEG analysis. Cai et al. (2018) distinguished depression patients from normal controls by fusing different EEG data sources, and the KNN classifier used achieved the highest classification accuracy of 86.98% after fusing multi-source data. Fan et al. (2020) used high-density 128-channel EEG and long and short-term memory network strategy based on convolution to diagnose depression, and the proposed model reached the accuracy of 83.47%. Aydemir et al. (2021) used wavelet and melamine pattern to extract features of EEG signals of patients with depression, and used KNN and SVM classifiers for classification to obtain high automatic recognition accuracy. Cai et al. (2020) proposed a new autism EEG signal conversion method, which used a combination of local binary patterns and short-time Fourier transform to generate the spectral features of the signal, and used a lightweight neural network for training, the resulting model can be used to aid in the diagnosis of autism. Most traditional research methods use 128-channel brain electrodes to collect as many brain channel signals as possible, which leads to huge computational complexity and is not conducive to real-time monitoring of depression patients. In recent years, many researchers have focused on the prefrontal brain, selecting FP1, FP2, and FPZ signals as the signal sources. Although certain effects have been achieved, there are still limitations. This is because the prefrontal data has fewer signal channels, which is more susceptible to data fluctuations. Meanwhile, EEG signals inherently contain a large number of redundant features. Therefore, removing as many EEG redundant features as possible while maintaining high real-time performance remains a highly challenging problem.

Therefore, the main innovations of this paper are as follows:

1) A sample entropy feature is proposed to describe the difference between EEG signals of depression patients and normal individuals. As an energy feature, entropy can effectively characterize the complexity changes of myoelectric signals.
2) A redundant signal elimination strategy combining Variational Mode Decomposition (VMD) and power spectrum is proposed. By decomposing EEG signals via VMD and selecting Intrinsic Mode Functions (IMFs) through power spectrum analysis, this approach helps eliminate redundant features in EEG signals. Combined with the LightBGM classification model, it achieves high accuracy and provides a feasible scheme for real-time monitoring of depression patients.

## 2 Materials and methods

### 2.1 Data description

**Figure 2:**
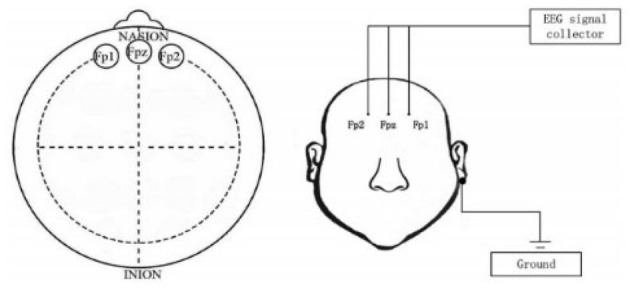
Location of frontal lobe EEG signal acquisition (Mohammadi and Moradi, 2021).

This experiment utilized a public dataset, namely the MODMA dataset (Mohammadi and Moradi, 2021), which was established by the Second Hospital of Lanzhou University. This dataset mainly consists of 55 participants, including a total of 26 outpatients diagnosed with depression (15 males and 11 females; aged 16–56 years), and 29 healthy controls (19 males and 10 females; aged 18–55 years). All MDD patients received a structured Mini-International Neuropsychiatric Interview (MINI) that met the diagnostic criteria for major depression of the Diagnostic and Statistical Manual of Mental Disorders (DSM) based on the DSM-IV. The dataset adopts a three-lead full-brain coverage EEG experimental protocol. According to the international 10–20 system electrode placement standard, three positioning points are selected on the forehead for electrode placement, with their specific pasting positions shown in the Figure 2. All subjects completed the Mini-Mental State Examination (MMSE) with the assistance of professional psychologists as a preliminary screening for depressive tendencies. If participants were at high risk of depression, they were required to additionally complete the Patient Health Questionnaire-9 (PHQ-9) to assess depression severity, while all basic information was collected. Candidate subjects were comprehensively determined to meet the experimental requirements based on self-rating scale data and inclusion criteria. Eligible subjects completed head cleaning under staff guidance and then wore detection equipment in a standard experimental environment. It should be specifically noted that: all subjects must not have taken any psychotropic drugs within 2 weeks before the experiment, and must not have other mental illnesses or organic brain injuries (such as epilepsy). Female subjects with depression must confirm that they are not pregnant. Meanwhile, the following conditions are excluded: lactating women, those taking contraceptives, individuals with a history of alcohol or psychotropic drug abuse/dependence within the past year, and individuals who have suffered abuse.

### 2.2 Sample entropy

Sample Entropy (SE) is a method proposed by Richman (2011) in 2000 to measure the complexity of time series. According to the principle and formula of sample entropy, a higher entropy value of a time series indicates greater complexity; conversely, a lower entropy value implies higher autocorrelation of the time series. In recent years, entropy has emerged as a novel method for evaluating the complexity and irregularity of EEG signals in individuals with Depressive Disorder. Increased EEG signal entropy has been observed in patients with DDD, which indicates enhanced complexity and reduced predictability of brain activity (Chen et al., 2020; Čukić et al., 2020). The integration of this information-theoretic approach is regarded as a promising method for the assessment and monitoring of clinical depression (Murphy et al., 2020).

1) A set of -dimensional vectors **X**(*q*) = {**X**(*q*), **X**(*q* + 1), ..., **X**(*q* + *k* − 1)}are constructed in order from a time series with a data quantity of *Q*, where *q* = 1, 2, ..., *Q* − *k* + 1.
2) Calculate the distance *d*_*ij*_ = max[|*x*(*i* + *g*) − *x*(*j* − *g*)|] between the *K*-dimensional vector **X**(*i*) and other vectors**X**(*j*), where *j* = 1, 2, ..., *Q* − *k* + 1, *g* = 0, 1, ..., *k* − 1, *i* ≠ *j*.
3) For a given sequence, define the number of *d*_*ij*_ ≤ *r*, (*r* > 0) as *B*_*i*_. The probability of matching *K* points is $B_{i}^{k}\left(r\right)$, whose mean is *B*^*k*^(*r*), and the formula is:
  (1) $$\begin{array}{l} B_{i}^{k}\left(r\right)=B_{i}/\left(Q-k+1\right) \end{array}$$
  (2) $$\begin{array}{l} B^{k}\left(r\right)=\frac{1}{Q-k+1}\overset{Q-k+1}{\underset{i=1}{\sum }}B_{i}^{k}\left(r\right) \end{array}$$
4) Increase the dimension *K* by 1, and repeat steps 1–3 to obtain *B*^*k*+1^(*r*) The estimated value of sample entropy is:
  (3) $$\begin{array}{l} \text{SampEn}\left(k,r,Q\right)=-\ln\;\left[B^{k+1}\left(r\right)/B^{k}\left(r\right)\right] \end{array}$$

In the formula: *r*– similarity tolerance.

### 2.3 Variational mode decomposition

Variational Mode Decomposition (Dragomiretskiy and Zosso, 2013) is a novel adaptive signal decomposition technique. It is a non-recursive method that decomposes a multi-component signal into an ensemble of band-limited intrinsic mode functions (IMFs), also known as modes or components, with specific sparsity properties.

The key advantages of VMD include its ability to adaptively decompose non-stationary and non-linear signals, its robustness to noise and sampling, and its capability to handle different types of signals, including those with closely spaced frequency components. VMD has found successful applications in various fields, such as biomedical signal processing, fault diagnosis, and financial time series analysis. The main steps of VMD are as follows:

1) The original signal *x*(*t*) can be directly defined as:
  (4) $$\begin{array}{l} x\left(t\right)=\overset{K}{\underset{k=1}{\sum }}u_{k}\left(t\right) \end{array}$$
2) For each mode function, the single-sided spectrum of the analysis signal can be obtained through Hilbert transform.
  (5) $$\begin{array}{l} \left[\delta \left(t\right)+\frac{j}{\pi t}\right]uk\left(t\right) \end{array}$$
  Where δ(*t*) is the Dirac function and *k* is the number of modes to be decomposed.
3) For each mode function *u*_*k*_(*t*), the basic frequency band after each modal spectrum modulation can be obtained by aliasing the exponential term $e^{-j\omega_{k}t}$of the center frequency ω_*k*_corresponding to the mode function *u*_*k*_(*t*).
  (6) $$\begin{array}{l} \left\{\left[\delta \left(t\right)+\frac{j}{\pi t}\right]u_{k}\left(t\right)\right\}e^{-j\omega kt} \end{array}$$
4) The bandwidth of each mode signal is estimated using the Gaussian smoothing method, which solves the variational problem under constraints. The objective function is:
  (7) $$\begin{array}{c} min_{\left\{u_{k}\right\},\left\{\omega_{k}\right\}}\{\sum k{\left\|\partial_{t}\{[\delta (\text{t})\text{+}\frac{j}{\pi t}]u_{k}(t)\}+e^{-j\omega kt}\right\|}^{2}\\ s.t.\sum_{k}u_{k}=f \end{array}$$
  Where {*u*_*k*_} = {*u*_1_, ……*u*_k_},{ω_*k*_} = {ω_1_, ……ω_k_},∂_*t*_ is the partial derivative with respect to *t*,*f*is the original complex signal before decomposition.
5) For the above variational problem, the solution process is as follow: The quadratic penalty factor α and the Lagrangian multiplication operator λ(*t*) are introduced into Equation 7 to transform the constrained variational problem into an unconstrained variational problem. The hyperparameter penalty factor α mainly ensures the reconstruction accuracy of the signal, while λ(*t*) maintains the strictness of the constraint conditions. Therefore, Equation 4 is expanded into the Lagrangian expression as follows:
  (8) $$\begin{array}{l} L\left(\left\{u_{\text{k}}\right\},\left\{\omega_{k}\right\},\lambda \right)=\alpha \underset{k}{\sum }∥\partial_{t}\left\{\left[\delta \left(t\right)+\frac{j}{\pi t}\right]u_{\text{k}}\left(t\right)\right\}\cdot e^{-j\omega_{k}t}∥\\ \;+<\lambda \left(\text{t}\right),\text{f}\left(\text{t}\right)-\underset{k}{\sum }u_{\text{k}}\left(\text{t}\right)> \end{array}$$

Use the alternating direction of the multiplier to calculate Equation 8 and continuously optimize by alternating and iteratively updating ${u_{\text{k}}}^{n+1}$, ${\omega_{\text{k}}}^{n+1}$ ,${\lambda_{\text{k}}}^{n+1}$ to obtain the optimal solution of Equation 8. Among them,${u_{\text{k}}}^{n+1}$ can be transformed into the frequency domain through Fourier transform, and we can get:


(9) $$\begin{array}{l} {\hat{u}}_{k}^{n+1}\left(\omega \right)=\underset{{\hat{u}}_{k},u_{k}\in X}{\arg\;\min}\{\alpha \left\|j\omega [[1+\text{sgn}(\omega +\omega_{k})]\cdot {\hat{u}}_{k}(\omega +\omega_{k})]\right\|\\ \;+{\left\|\overset{\wedge }{\;f}(\omega )-\sum_{i}{\hat{u}}_{i}(\omega )+\frac{\overset{\wedge }{\lambda }(\omega )}{2}\right\|}_{2}^{2}\} \end{array}$$

Where *X* is the constraint condition of û_*k*_, *u*_*k*_ that is, $\underset{k}{\sum }u_{k}=f$; the purpose of the quadratic penalty factor α is mainly to reduce the signal interference of Gaussian noise;$\overset{\wedge }{\lambda }(\omega )$ is the tolerance of the entire noise signal, which is mainly used to ensure that the signal after decomposition is not distorted; $\overset{\wedge }{\;f}(\omega )$ is the Fourier transform of $f(\omega ),{\hat{u}}_{i}(\omega )$ is the Fourier transform of u_*k*_(*t*).

Equation 9 can be transformed into the frequency domain through Fourier transform, and then the solution of ${{\hat{u}}_{k}}^{n+1}\left(\omega \right)$ can be obtained as:


(10) $$\begin{array}{l} {\hat{u}}_{k}^{n+1}\left(\omega \right)=\frac{\hat{f}\left(\omega \right)-\underset{i}{\sum }{\hat{u}}_{i}\left(\omega \right)+\frac{\hat{\lambda }\left(\omega \right)}{2}}{1+2\alpha {\left(\omega -\omega_{k}\right)}^{2}} \end{array}$$

In our proposed method, VMD is used to decompose the EEG signal into its intrinsic mode or component, which can then be used for component selection using the power spectrum, avoiding interference from redundant information.

### 2.4 LightGBM

Light Gradient Boosting Machine (LightGBM) is a highly efficient implementation of the gradient boosting decision tree (GBDT) algorithm, proposed by Ke et al. to address the limitations of traditional GBDT in handling large-scale datasets, such as high computational complexity and slow training speed [1]. Distinguished by two core optimization strategies–Gradient-based One-Side Sampling (GOSS) and Exclusive Feature Bundling (EFB)–LightGBM achieves significant improvements in training efficiency while maintaining or enhancing prediction accuracy, making it widely applied in fields like machine learning, data mining, and biomedical signal analysis (e.g., EEG-based depression detection).

#### 2.4.1 Gradient-based one-side sampling (GOSS)

GOSS focuses on sampling instances with large gradients (critical for model update) while retaining a small proportion of instances with small gradients to preserve the overall data distribution. Specifically, during each iteration:

a. Sort training instances by the absolute value of their gradients in descending order.
b. Select the top a × 100% instances (large-gradient samples) as core samples.
c. Randomly sample b × 100% instances from the remaining (1-a) × 100% instances (small-gradient samples) and multiply their gradients by a weight factor $\frac{1-a}{b}$ to compensate for the sampling bias.

#### 2.4.2 Objective function

The objective function of LightGBM follows the gradient boosting framework, combining a loss function and a regularization term to prevent overfitting. For the t-th iteration, the objective function is defined as:


(11) $$\begin{array}{l} O^{\left(t\right)}=\overset{n}{\underset{i=1}{\sum }}L\left(y_{i},{\hat{y}}_{i}^{\left(t-1\right)}+f_{t}\left(x_{i}\right)\right)+\Omega \left(f_{t}\right) \end{array}$$

where n is the number of training instances, y_*i*_ is the true label of the i-th instance, ${\hat{y}}_{i}^{\left(t-1\right)}$ is the predicted value of the i-th instance after *t* − 1 iterations, Ω(*f*_*t*_) is the regularization term for the t-th tree, defined as:


(12) $$\begin{array}{l} \Omega \left(f_{t}\right)=\gamma T+\frac{1}{2}\lambda \overset{T}{\underset{j=1}{\sum }}w_{j}^{2} \end{array}$$

Here, T is the number of leaves in the t-th tree, ω_*i*_ is the score of the j-th eaf, and γ, λ are regularization parameters.

### 2.5 Proposed recognize model

**Figure 3:**
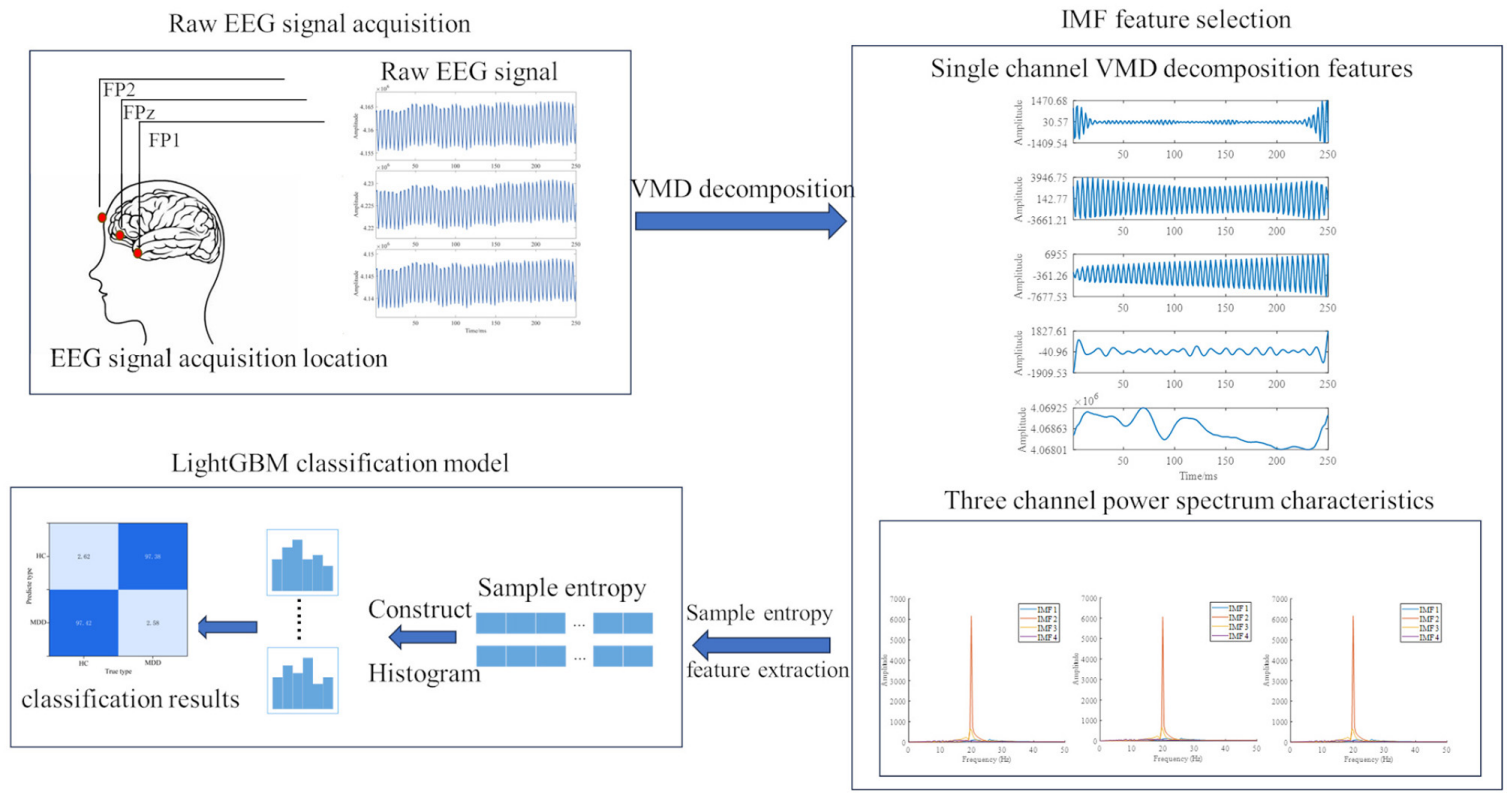
Model framework structure diagram.

**Figure 4:**
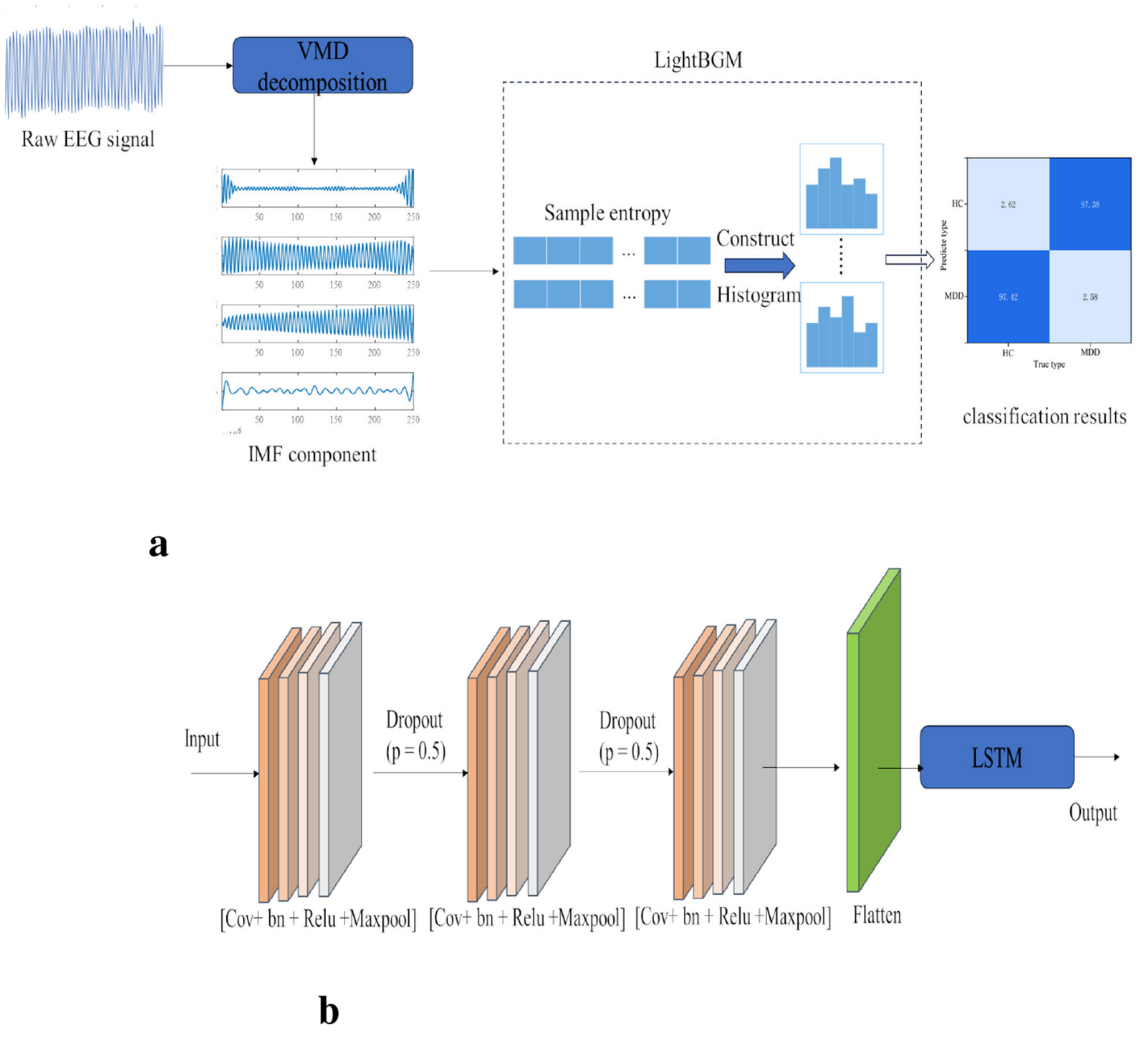
Model framework diagram. **(a)** framework diagram of the vmd-lightgbm model. **(b)** 3-layer CNN-LSTM model framework diagram.

The algorithm flowchart selected in this paper is shown in Figure 3. First, three-channel EEG signals are collected through the human prefrontal lobe. The collected signals undergo feature extraction via Variational Mode Decomposition (VMD), and appropriate feature components are calculated by combining the power spectrum. Finally, sample entropy is calculated for the obtained feature components. After obtaining the entropy features, they are fed into the LightBGM network for classification to ultimately determine whether the subject is in a depressive state or a depressive patient. The block diagram of its algorithm is shown in the Figure 4a.

As can be seen from Figure 4a, after inputting the original EEG signals, they are first decomposed using Variational Mode Decomposition (VMD) to obtain a total of 4 Intrinsic Mode Function (IMF) feature components. These four components undergo sample entropy feature extraction, and the resulting features are finally input into LightGBM for classification.

## 3 Experiments

### 3.1 VMD signal decomposition and IMF component selection

**Figure 5:**
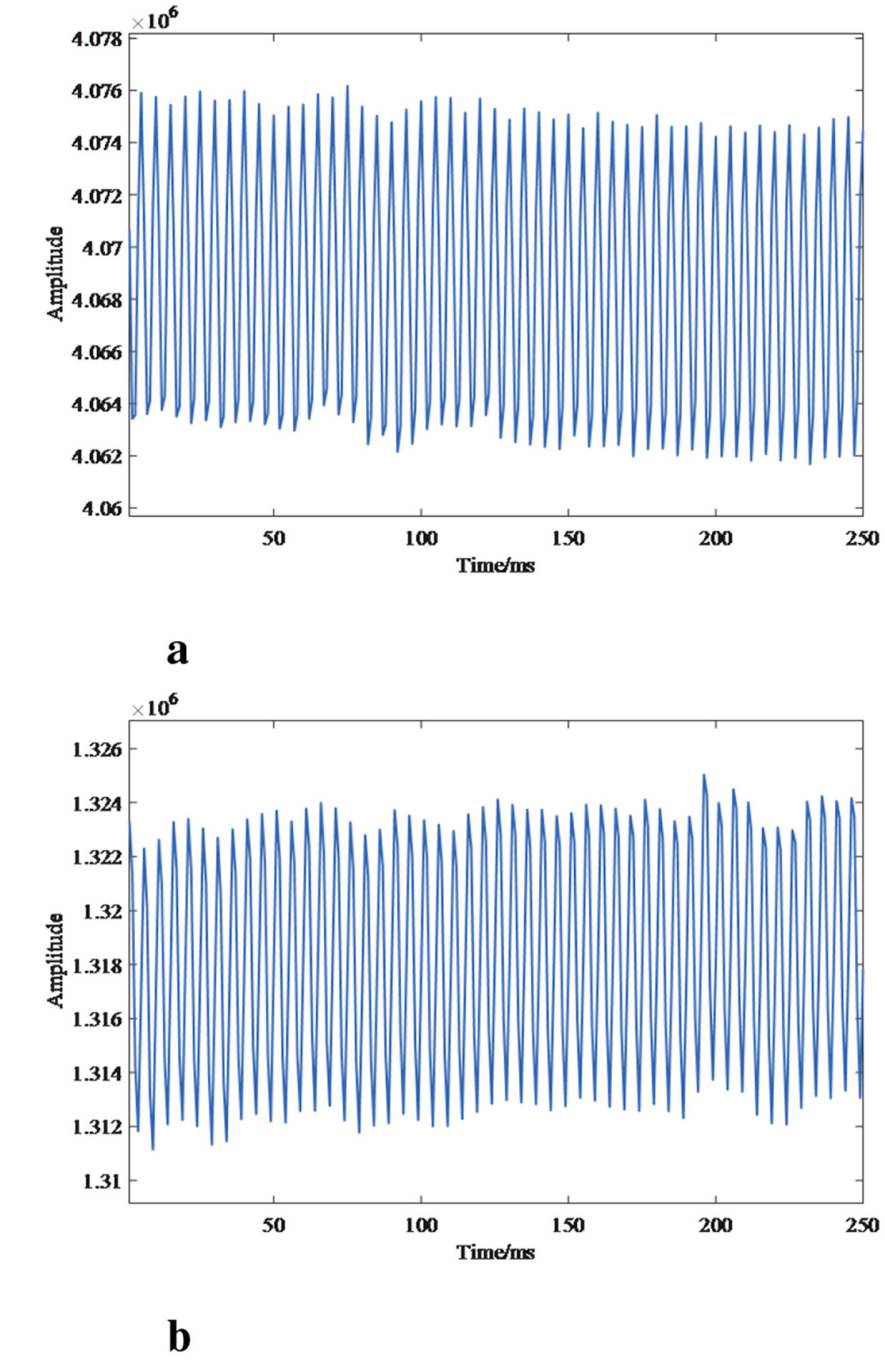
EEG data of HC and MDD individuals **(a)** HC data. **(b)** MDD data.

Considering the strong correlation between the prefrontal lobe and emotional processes, as well as mental illnesses, electroencephalogram (EEG) signals were collected via three electrodes. A common EEG acquisition device has three electrodes (Fp1, Fpz, and Fp2) on the prefrontal lobe. Data were recorded in a room free of loud noise and strong magnetism. Participants kept their eyes closed until their EEG signals were observed to be relatively stable, after which we began 90-s data acquisition, the sampling frequency is 250 Hz. In the processing of the MODMA dataset, the original hexadecimal data were first converted into decimal data. Then, the signals were filtered with a 1 Hz high-pass and 45 Hz low-pass finite impulse response (FIR) filter. Finally, the dataset used an adaptive noise canceller to eliminate eye-blink artifacts, thereby obtaining the noise-removed EEG signal data. The resulting EEG signals are shown in Figure 5, where HC represents healthy subjects and MDD represents major depressive disorder patients.

**Figure 6:**
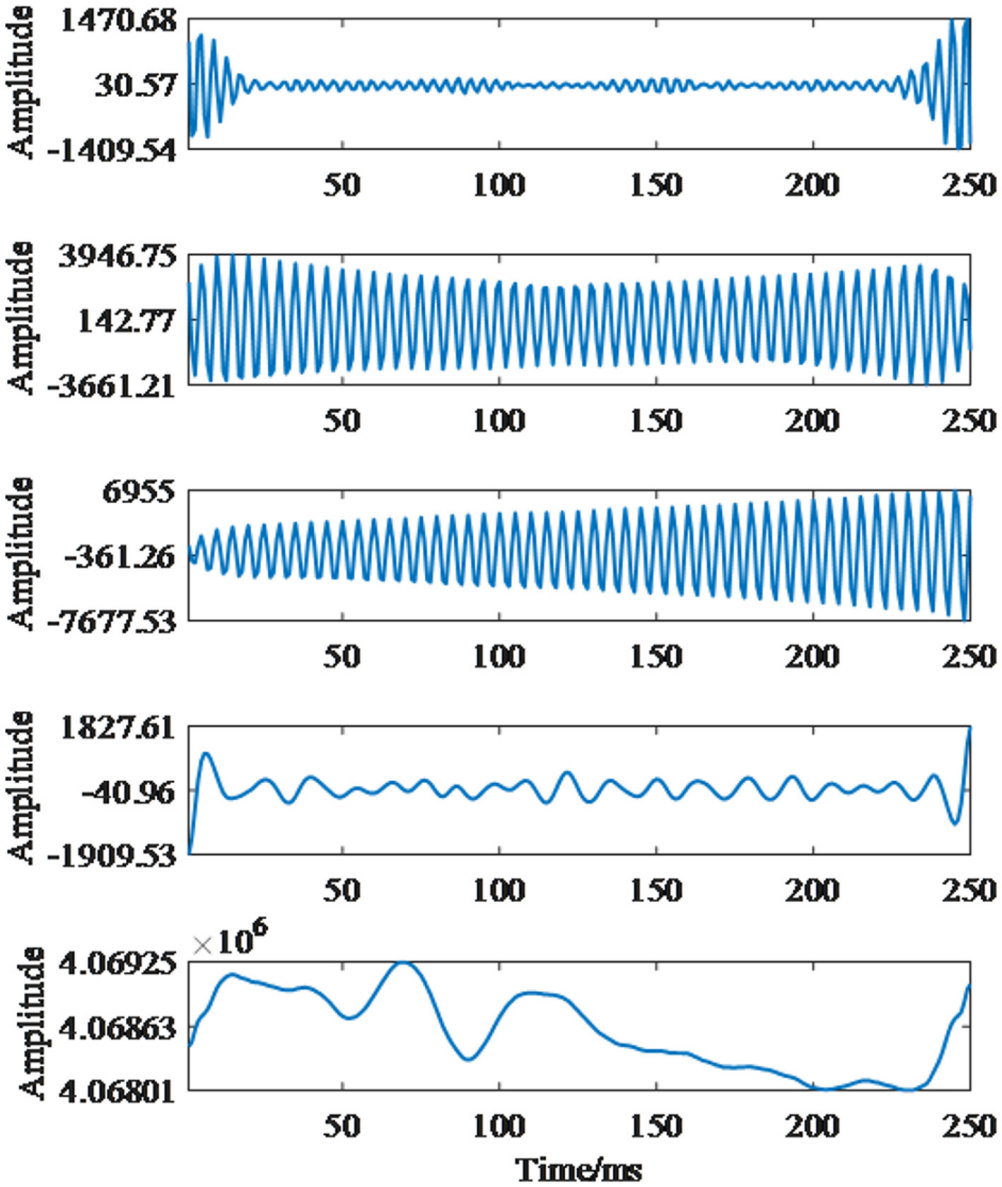
EEG signal VMD decomposition results.

First, the data of each group were decomposed by VMD. The Variational Mode Decomposition (VMD) method is adopted mainly because the EEG activities in the δ, θ, α, and β frequency bands of patients with depression are generally higher than those of the normal control group. Moreover, the α and β frequency bands contain more depression-related EEG information than the low-frequency δ and θ bands. Therefore, decomposing the EEG signal into different frequency bands via VMD can effectively filter out interference from other frequency bands and ensure the validity of the signal. The VMD technique was used to decompose the non-stationary EMG signals into multiple frequency-band-limited IMFs, making each decomposed component easier to distinguish for emotion state classification. Taking the HC data as an example, parameter enumeration and optimization of the VMD algorithm were performed to obtain five groups of components. The decomposition results are shown in Figure 6. The characteristic components after VMD decomposition can more obviously reflect the variation trends of EEG signals at different frequencies, and the variation characteristics of EEG can be retained by extracting the effective fluctuation information of each modal component.

**Figure 7:**
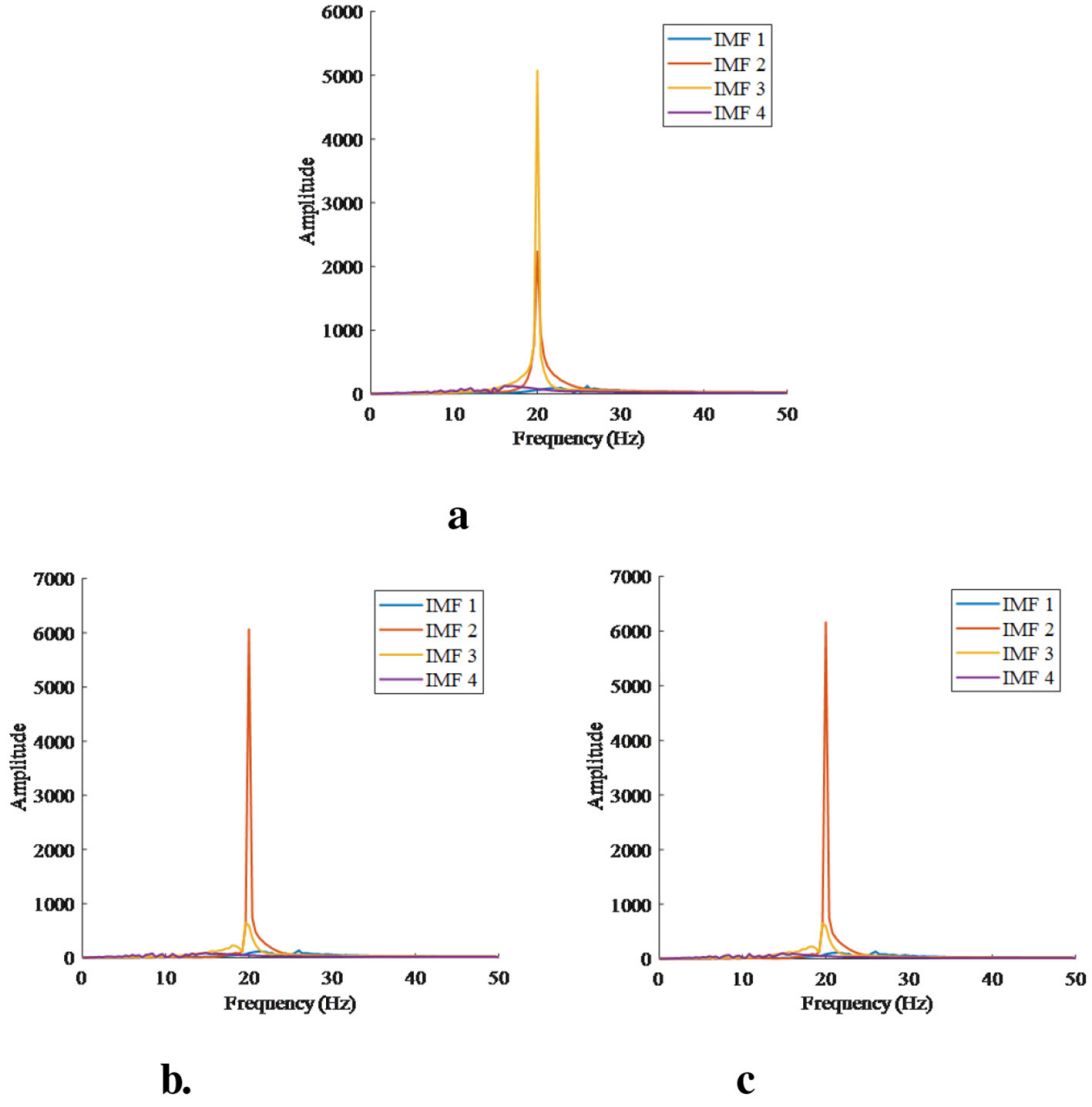
Power spectrum analysis of EEG three channel signals using IMF. **(a)** Channel1. **(b)** Channel2. **(c)** Channel3.

In this work, frequency analysis was conducted to determine the primary IMFs. Figure 7 shows the sample power diagrams of the primary IMFs. Since the frequency distribution of IMF5 differs significantly from that of the remaining components, only IMF1-IMF4 were selected as the primary IMF components for feature extraction. Additionally, it can be clearly observed from the power spectrograms that although the signal is decomposed into multiple groups of IMFs, highly discriminative information is retained only in a few IMFs. IMF2 and IMF3 exhibit higher energy density, while IMF1 and IMF4 have relatively lower energy content.

After obtaining the IMFs, we extracted sample entropy features from the main IMFs. This feature can effectively characterize the changes in EEG complexity and avoid Gaussian noise interference. After extracting the sample entropy features, since there are three channels in total, each channel is decomposed into 4 IMF channels, and sample entropy features are extracted, we finally obtained 12 feature vectors.

### 3.2 Influence of learning rate on model accuracy

To ensure the accuracy of evaluation results, this paper uses Accuracy, Precision, Recall, F1-Score, Confusion Matrix, and average time consumption (T) as evaluation indicators for the lower limb gait phase recognition method. Here, TP represents the number of samples correctly predicted as positive by the model, FN represents the number of positive samples incorrectly predicted as negative by the model, FP represents the number of samples incorrectly predicted as positive by the model, and TN represents the number of samples incorrectly predicted as negative by the model.


(13) $$\begin{array}{l} Accuracy=\frac{TP+TN}{TP+TN+FP+FN} \end{array}$$

(14) $$\begin{array}{l} Precision=\frac{TP}{TP+FP} \end{array}$$

(15) $$\begin{array}{l} Recall=\frac{TP}{TP+FN} \end{array}$$

(16) $$\begin{array}{l} F1-score=\frac{2\cdot Precision\cdot Recall}{Precision+Recal} \end{array}$$

The average recognition time evaluates the real-time performance of the model by calculating the recognition time of each sample.


(17) $$\begin{array}{l} T=\frac{1}{n}\overset{n}{\underset{i}{\sum }}t_{i},i=1,2,...,N \end{array}$$

To optimize the parameters of the LightGBM classifier model, an enumeration method was used to search for optimal parameter combinations of different numbers of leaves and learning rates, thereby determining the best values of hyperparameters. The influence of model hyperparameters on the classifier's accuracy is shown in Table 1. It can be seen from the table that the highest classification accuracy is achieved with the parameters of a maximum number of leaves of 50 and a learning rate of 0.01. Therefore, this set of hyperparameters will be used for subsequent model training and testing.

**Table 1:** Model accuracy results under different hyperparameters.

	**Number of leaves**					
**LR**	**10.0**	**20.0**	**30.0**	**40.0**	**50.0**	**100.0**
0.01	94.98	95.68	96.39	96.45	97.78	97.28
0.005	94.15	95.54	96.44	96.78	97.56	97.16
0.001	92.82	94.45	95.38	95.87	96.45	97.05
0.0005	92.68	93.87	94.96	95.22	96.35	97.04
0.0001	92.69	94.21	94.54	95.12	96.15	96.85

To verify the generalization ability of the model, the test set data without cross-validation was used for model inspection, and the model accuracy is shown in Table 2.

**Table 2:** 5 fold cross validation model testing accuracy.

**Indicator parameters**	**Acc/%**	**Pre/%**	**Re/%**	**F1/%**
Flod 1	97.56 ± 0.31	97.19 ± 0.28	97.38 ± 0.24	97.43 ± 0.21
Flod 2	97.35 ± 0.27	97.23 ± 0.22	97.24 ± 0.21	97.31 ± 0.19
Flod 3	97.27 ± 0.23	97.16 ± 0.17	97.25 ± 0.18	97.18 ± 0.20
Flod 4	97.34 ± 0.28	97.21 ± 0.23	97.32 ± 0.25	97.26 ± 0.22
Flod 5	97.58 ± 0.26	97.46 ± 0.21	97.48 ± 0.23	97.43 ± 0.22
Average	97.42 ± 0.27	97.25 ± 0.22	97.33 ± 0.22	97.33 ± 0.21

**Figure 8:**
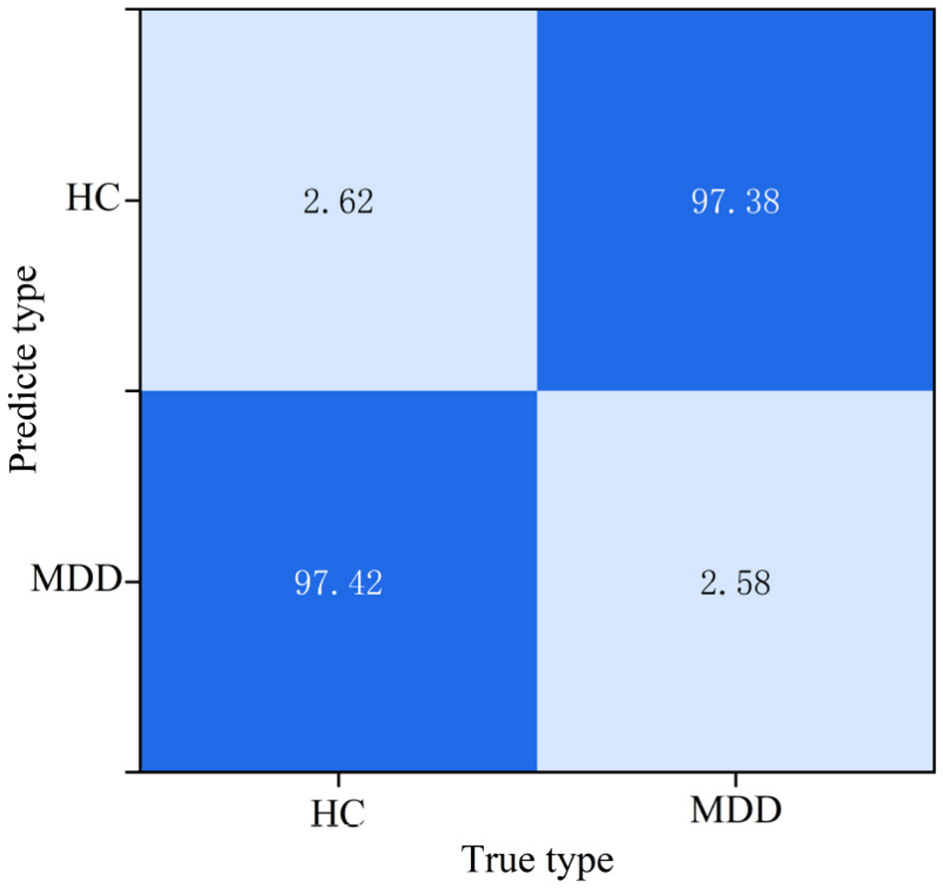
Classification confusion matrix.

Combined with Table 2, it can be seen that after 5-fold cross-validation, the accuracy of the model adopted in this paper on each fold of the test set remains above 97.27%, with an average recognition accuracy of 97.42%. Among them, the fifth fold of data achieves the highest accuracy, while the third fold of data has relatively the lowest accuracy. The model exhibits good stability, and the accuracy on the test set can distinguish data of different categories, further verifying the effectiveness of the model. To fully exploit and utilize the temporal features in the training set and enhance the model's generalization ability, the trained model is applied to the test set for evaluation, and the confusion matrix shown in Figure 8 is obtained. The confusion matrix can intuitively reflect the classification performance of the model under various categories, where HC represents healthy subjects and MDD represents major depressive disorder patients.

As shown in Figure 8, the recognition accuracy of each phase in the test set is above 97.42%. During the learning process, the model needs to effectively extract and classify features from a large number of complex and interleaved features, but the overall recognition accuracy is high. Similar features in some data may lead to misjudgments. The experimental results in this paper show that the model can accurately determine the emotional state of the population, providing a universal judgment basis for related applications. Therefore, the effectiveness of the model using EEG signals for depression recognition is verified.

### 3.3 Comparative experiments

To further verify the effectiveness and superiority of the proposed method, this subsection conducts horizontal comparisons using algorithms that have achieved excellent results in emotion recognition and pattern recognition in recent years. The comparative methods cover both machine learning and deep learning approaches, all trained and tested on the same dataset. The comparison methods cover machine learning and deep learning methods, all of which are trained and tested on the same dataset. In terms of dataset division, we perform a sliding window on 90-s data samples with a length of 1 s, resulting in 4,950 samples. The dataset is segmented at 1-s intervals, mainly because there are many factors that ultimately affect the occurrence of MUS. A large proportion of these factors are caused by somatization and depression. This paper only discusses the technical means for preliminary screening of depression. With only one second of data, it can effectively determine whether an individual suffers from depression, thereby realizing the front-end triage of undifferentiated disorders, quickly screening out depressed patients from normal individuals, and improving the diagnostic efficiency. However, due to certain feature similarity between adjacent sliding windows, using the conventional 7:3 random division of samples in machine learning may easily lead to similarity between some samples in the test set and training set. Therefore, we divide the 4,950 samples according to the time dimension, with the first 50% of the samples as the training set and the last 50% as the test set. From the training set, 70% is extracted for training and 30% for validation. To ensure the effectiveness of the model, all input sample data undergo VMD component decomposition and sample entropy feature extraction. In this study, we used a laptop with an Intel i5-12400F @2.5 GHz CPU and an NVIDIA RTX 3060 GPU as the hardware environment. The software environment consists of Python with PyTorch 2.2.2. The algorithm comparison results are shown in Table 3.

**Table 3:** Comparison results of accuracy performance of different recognition methods.

**Method**	**Accuracy/%**	**Precision/%**	**Recall/%**	**F1-Score/%**	**T/ms**	**Para/M**
SVM	89.29 ± 0.01	89.71 ± 0.01	89.32 ± 0.01	89.19 ± 0.01	1.86	0.24
RF	90.68 ± 0.02	90.67 ± 0.02	90.68 ± 0.02	90.71 ± 0.02	1.52	0.25
LightGBM	93.78 ± 0.02	93.62 ± 0.03	93.72 ± 0.01	93.64 ± 0.02	0.06	0.02
2CNN-LSTM	95.23 ± 0.24	95.18 ± 0.35	95.26 ± 0.24	95.13 ± 0.36	2.52	2.74
3CNN-LSTM	97.12 ± 0.34	97.15 ± 0.32	97.12 ± 0.32	97.10 ± 0.36	2.82	3.18
2CNN - BiLSTM	93.89 ± 0.44	93.77 ± 0.46	93.87 ± 0.38	93.69 ± 0.48	2.36	2.87
3CNN - BiLSTM	97.72 ± 0.54	97.77 ± 0.59	97.72 ± 0.54	97.73 ± 0.56	2.90	3.38
MACNN	96.78 ± 0.34	96.53 ± 0.32	96.51 ± 0.21	96.47 ± 0.33	2.80	3.15
Our method	97.42 ± 0.27	97.25 ± 0.22	97.33 ± 0.22	97.33 ± 0.21	2.23	0.32

For the CNN-LSTM model, in terms of network structure, we adopted a network structure where a 3-layer CNN network is connected in series with LSTM. Here, the “3 layers” refer to 3 modules, and each module includes a 3 × 3 convolution, regularization, a ReLU layer, and a global pooling layer, as shown in Figure 4b. We chose to use the Adam optimizer, set the learning rate to 0.001, set the total number of epochs to 100, and set the batch size to 64. The loss function used is the cross-entropy loss function. For the CNN-BiLSTM model, we only performed bidirectional processing on the LSTM model, and the remaining parameters are the same as those of the CNN-LSTM model. The experimental results show that the method proposed in this paper achieves the best performance in classification, with an average recognition accuracy of 97.42%, significantly superior to other comparative algorithms. In terms of model parameters, this method only has 0.32 M parameters, far lower than other deep learning algorithms, demonstrating its lightweight advantage.

In machine learning methods, relying on manually extracted features inevitably leads to partial loss of EEG features, directly limiting the model accuracy of traditional algorithms such as SVM and RF. However, the machine learning algorithm LightGBM, with its efficient decision tree mechanism, can not only achieve high classification accuracy but also maintain extremely small model parameters under the same EEG feature input, reflecting the effectiveness of lightweight models in feature utilization.

**Figure 9:**
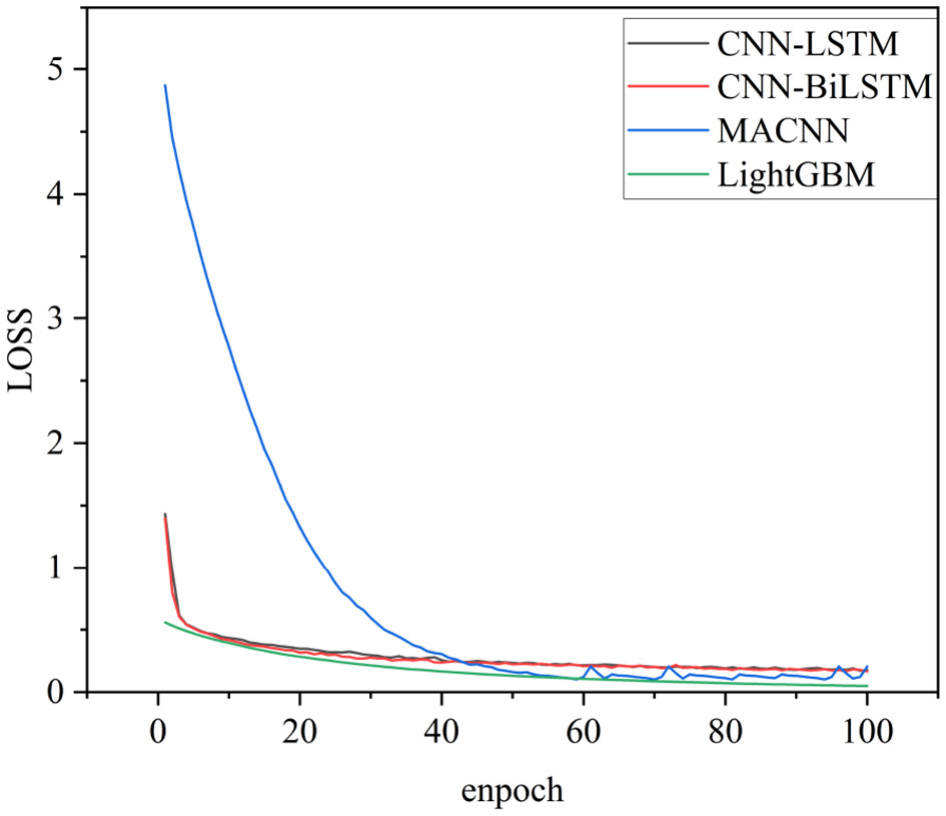
Training loss curves of CNN-LSTM, CNN-BiLSTM and LightGBM.

In the field of deep learning, 3-layerCNN-LSTM and 3-layerCNN-BILSTM models improve classification accuracy compared with traditional machine learning methods by fusing the temporal features of EEG signals, which verifies the critical impact of signal temporal information on classification performance. The training loss is shown in Figure 9. It can be seen from Figure 7 that Multi-Attention Convolutional Neural Network (MACNN) converges relatively slower than both CNN-LSTM and the proposed algorithm in this paper, and its final accuracy is also lower than the proposed algorithm. Meanwhile, in terms of time consumption, the MACNN algorithm takes longer. However, the proposed algorithm further breaks through the limitation of a single feature dimension through a multi-scale feature extraction strategy. In horizontal comparison, although the performance effect is slightly lower than that of 3CNN-Bilstm, it is stronger than 3CNN-Bilstm in terms of model lightweight and computational time. In summary, the proposed method shows significant advantages in three dimensions: classification accuracy, number of parameters and recognition time, and provides a more practical solution for the classification of depression based on EEG signals.

**Figure 10:**
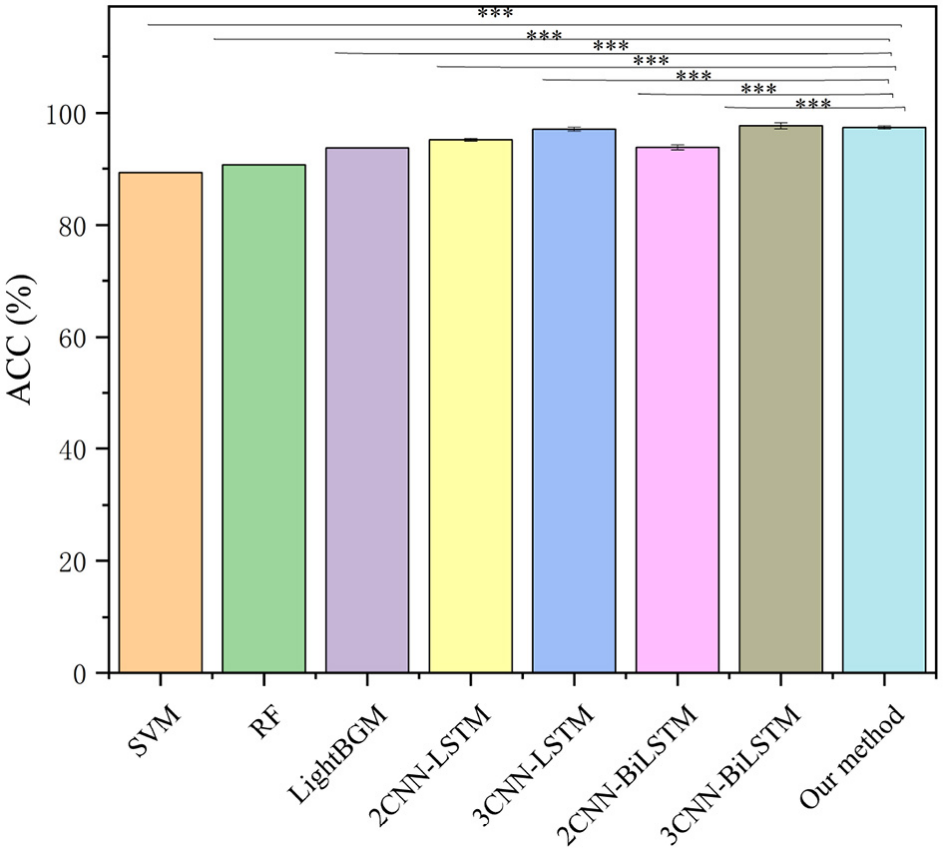
Significance testing and comparative results of different recognition methods. Significance markers denote statistical differences (paired *t*-test): ^***^p < 0.001.

A one-way analysis of variance was used to measure the significant difference level between the comparative methods and the proposed method. As shown in Figure 10, there are significant differences (*p* ≤ 0.001) between the proposed method and other comparative methods, These comparison algorithms have lower model recognition accuracy than the proposed method on the same dataset, with only 3CNN-BiLSTM being slightly higher than the algorithm in this paper by 0.3%. However, as can be seen from Table 3, the computation time of 3CNN-BiLSTM is 2.9 ms, while that of the algorithm in this paper is only 2.23 ms. Considering both accuracy and timeliness, the algorithm in this paper has certain effectiveness in this classification task.

### 3.4 Ablation and feature selection experiments

To further screen the influence of features and verify the effectiveness of each layer in the proposed model, we conducted ablation experiments, and the results are shown in Table 4.

**Table 4:** Results of ablation experiments and feature selection.

**Indicator parameters**	**Acc/%**	**Pre/%**	**Re/%**	**F1/%**
lightGBM +RawData	93.78 ± 0.02	93.62 ± 0.03	93.72 ± 0.01	93.64 ± 0.02
lightGBM +VMD	95.27 ± 0.25	95.32 ± 0.22	95.18 ± 0.20	95.23 ± 0.18
lightGBM +VMD + Sample entropy	97.42 ± 0.27	97.25 ± 0.22	97.33 ± 0.22	97.33 ± 0.21
lightGBM +VMD + RMS	96.58 ± 0.18	96.37 ± 0.21	96.41 ± 0.15	96.53 ± 0.16
lightGBM +VMD + PSD	96.28 ± 0.16	96.18 ± 0.17	96.16 ± 0.14	96.13 ± 0.12

In terms of dataset division, to avoid the situation where the dataset has high feature similarity due to sliding windows, we divided the entire sample into two parts in a ratio of 5:5. The first 50% of the total sample dataset is the training set, and the latter 50% is the test set. Meanwhile, we extracted 70% from the training set for model training, and 30% from the test set as the test set for the model. First, we used the original EEG data without any feature extraction and input it into the lightGBM model, achieving an accuracy of 93.78%. This is because there is a certain difference between patients and healthy people in binary classification data. Then, we added VMD for modal decomposition of the model, where *K* = 4. Each channel obtained 4 modal decomposition vectors, and then 12-dimensional features were input into the model for classification. Multiple measurements showed a classification accuracy of 95.27%. Finally, after adding the sample entropy, the overall accuracy increased by 2.15%, which proves the effectiveness of the sample entropy feature.

To further verify the effectiveness of the features, we selected three common features, namely the sample entropy feature, RMS, and PSD feature. After VMD decomposition into 12-dimensional vector data, we performed a sliding window with a window length of 20 data points, extracted feature vectors, and input them into the model for recognition. Tests showed that the sample entropy feature was slightly higher than the RMS and PSD features, thus proving the effectiveness of the model.

## 4 Discussion

Generalized undifferentiated symptoms refer to pain, fatigue, gastrointestinal, cardiovascular, and other symptoms that cannot be fully explained by MUS, which are very common in the elderly population. Combined with case analysis, we found that these medically unexplainable phenomena often occur in middle-aged and elderly people, mainly caused by mental illnesses, with depression being the most common condition.

Currently, the main medical diagnostic tool for depression is psychological scales, which are highly subjective. Additionally, patients' resistance to psychological scales leads to inaccurate judgments. Therefore, EEG signals, as a more objective evaluation criterion, have been widely used in the diagnosis of mental diseases. Conventional detection using 128-channel EEG signals provides relatively complete data features but cannot meet real-time detection requirements. Considering the strong correlation between the prefrontal lobe and emotional processes as well as mental illnesses, three electrodes (Fp1, Fpz, and Fp2) were selected on the prefrontal lobe for measurement. This significantly reduces data volume and improves calculation speed, but the collected data may suffer from decreased judgment accuracy due to incomplete features. Therefore, a new algorithm is needed to improve classification accuracy.

In terms of feature extraction, we chose to decompose EEG signals using VMD and optimized parameters through enumeration to obtain five groups of components as shown in the figures. Since the frequency distribution of IMF5 differs significantly from the remaining components, only IMF1-IMF4 were selected as the main IMF components for feature extraction. Power spectral density was calculated for these five groups of components, revealing that effective feature components are stored in a small number of IMF components. Therefore, we selected the first four components as input features, resulting in a total of 12 groups of feature vectors from three channels. Sample entropy was calculated for these feature vectors to obtain the input features for the model.

After inputting the features into the LightGBM classification model, we considered the impact of learning rate and number of leaves on model classification accuracy. As shown in Table 1, the model achieved the highest classification accuracy with a maximum number of leaves set to 50 and a learning rate of 0.01. To further verify the model's generalization ability, Table 2 shows that after 5-fold cross-validation, the accuracy of the model proposed in this paper on each fold of the test set remained above 97.58%, with an average recognition accuracy of 97.42%. Among them, the fourth fold of data achieved the highest accuracy, while the first fold had relatively the lowest accuracy. Therefore, the proposed model exhibits good stability, and its accuracy on the test set can distinguish data of different categories, further verifying the model's effectiveness.

In the comparative experiments, we selected mainstream machine learning and deep learning algorithms for comparison. The experimental results in Table 3 show that the proposed method achieves the best classification performance, with an average recognition accuracy of 97.42% and a total time consumption of 2.23 ms. Considering both timeliness and accuracy, it is superior to other algorithms. Although deep learning models have slightly higher accuracy, their complexity leads to longer time consumption. Therefore, to realize engineering applications, the lightweight algorithm proposed in this paper has high application value.

Therefore, the classification model proposed in this paper balances accuracy and real-time performance, and is superior to other common depression detection algorithms, providing a solid foundation for the application of EEG signals in depression emotion detection. In this paper, 1 s of EEG data is used for pre-triage of patients with Medically Unexplained Symptoms to rule out psychological factors such as somatization and depression, which can effectively improve the efficiency of medical diagnosis. Meanwhile, the algorithm proposed in this paper enhances the real-time performance of detection. Although individual differences may lead to a slight decrease in the accuracy of the algorithm, as a front-end module for pre-triage, it provides a solution for EEG signal devices in real-time depression detection and pre-triage of MUS patients.

## 5 Conclusions

MUS is one of the emerging fields in current research. Among middle-aged and elderly patients, most MUS symptoms are mainly caused by depression. However, because the symptoms do not meet the international diagnostic criteria for depression somatization, doctors cannot make an effective judgment on depression. This may delay treatment time, thereby exacerbating depression and threatening lives. In current research, many scholars hope to judge whether one suffers from depression through EEG signals. However, due to the complexity of EEG signals, their susceptibility to noise pollution, the need for a large number of channels to collect, and the long computation time, the application of EEG in depression diagnosis is limited. To improve the applicability of EEG in the diagnosis of depression, this paper proposes a deep learning model for diagnosing depression using three-channel electroencephalogram (EEG) signals. The signal is decomposed by variational mode decomposition (VMD), and the number of intrinsic mode functions (IMFs) is determined by power spectrum analysis, thereby enhancing the feature dimension of the model. Sample entropy is used to extract features from the collected information, and a classification accuracy of 97.42% is finally achieved. Through 5-fold cross-validation, the model is significantly superior to other traditional algorithms, demonstrating certain generalization ability.

The fast detection algorithm proposed in this paper uses only 3 channels. While pursuing high timeliness, it acquires a small amount of data and contains a small number of EEG features. To achieve high classification accuracy, we use the VMD algorithm for decomposition, thereby upgrading the 3-channel data to 12 dimensions, and use sample entropy for feature extraction to increase the feature dimension of the signal, thus achieving high classification accuracy. This strategy further breaks through the limitation of a single feature dimension, achieves the best recognition performance in horizontal comparison, and balances the requirements of model stability and lightweight. Therefore, the algorithm proposed in this paper provides a solution for real-time depression monitoring using EEG signal equipment.

## Data Availability

The original contributions presented in the study are included in the article/supplementary material, further inquiries can be directed to the corresponding author.

## References

[B1] Akbari H. Sadiq M. Payan M. Esmaili S. Baghri H. Bagheri H. (2021). Depression detection based on geometrical features extracted from sodp shape of eeg signals and binary pso. Traitement Du Signal 38:13–26. 10.18280/ts.38010236359630

[B2] Alhalaseh R. Alasasfeh S. (2020). Machine-learning-based emotion recognition system using EEG signals. Computers 9:95. 10.3390/computers9040095

[B3] Aydemir E. Tuncer T. Dogan S. Gururajan R. Acharya U. (2021). Automated major depressive disorder detection using melamine pattern with eeg signals. Appl. Intell. 51, 6449–6466. 10.1007/s10489-021-02426-y

[B4] Bachmann M. Paeske L. Kalev K. Aarma K. Lehtmets A. Oopik P.. (2018). Methods for classifying depression in single channel EEG using linear and nonlinear signal analysis. Comput. Methods Programs Biomed. 155, 11–17. 10.1016/j.cmpb.2017.11.023 29512491

[B5] Barsky A. Orav E. Bates D. (2005). Somatization increases medical utilization and costs independent of psychiatric and medical comorbidity. Arch. Gen. Psychiatry 62, 903–910. 10.1001/archpsyc.62.8.903 16061768

[B6] Berger C. Dueck A. Perin F. Wunsch K. Buchmann J. Kolch M.. (2021). Brain arousal as measured by EEG-assessment differs between children and adolescents with attention-deficit/hyperactivity disorder (ADHD) and depression. Front. Psychiatry 12:633880. 10.3389/fpsyt.2021.633880 34777030 PMC8581225

[B7] Cai H. Chen Y. Han J. Zhang X. Hu B. (2018). Study on feature selection methods for depression detection using three-electrode EEG data. Interdiscip. Sci. Comput. Life Sci. 10, 558–565. 10.1007/s12539-018-0292-5 29728983

[B8] Cai H. Gao Y. Sun S. Li N. Hu B. (2020). MODMA dataset: a multi-model open dataset for mental- disorder analysis. arXiv preprint arXiv:2002.09283.

[B9] Cao Z. Lin C. Ding W. Chen M. Li C. Su T. (2019). Identifying ketamine responses in treatment-resistant depression using a wearable forehead EEG. IEEE Trans. Biomed. Eng. 66, 1668–1679. 10.1109/TBME.2018.2877651 30369433

[B10] Chen F. Zhao L. Li B. Yang L. (2020). Depression evaluation based on prefrontal EEG signals in resting state using fuzzy measure entropy. Physiol. Meas. 41:95007. 10.1088/1361-6579/abb144 33021227

[B11] Claassen-van Dessel N. van der Wouden J. Hoekstra T. Dekker J. van der Horst H. (2018). The 2-year course of medically unexplained physical symptoms (mups) in terms of symptom severity and functional status: results of the prospects cohort study. J. Psychosom. Res. 104, 76–87. 10.1016/j.jpsychores.2017.11.012 29275789

[B12] Creed F. Barsky A. (2004). A systematic review of the epidemiology of somatisation disorder and hypochondriasis. J. Psychosom. Res. 56, 391–408. 10.1016/S0022-3999(03)00622-6 15094023

[B13] Čukić M. Stokić M. Simić S. Pokrajac D. (2020). The successful discrimination of depression from EEG could be attributed to proper feature extraction and not to a particular classification method. Cogn. Neurodyn. 14, 443–455. 10.1007/s11571-020-09581-x 32655709 PMC7334335

[B14] De la Salle S. Choueiry J. Shah D. Bowers H. McIntosh J. Ilivitsky V.. (2016). Effects of ketamine on resting-state EEG activity and their relationship to perceptual/dissociative symptoms in healthy humans. Front. Pharmacol. 7:348. 10.3389/fphar.2016.00348 27729865 PMC5037139

[B15] de Waal M. Arnold I. Eekhof J. van Hemert A. (2004). Somatoform disorders in general practice: prevalence, functional impairment and comorbidity with anxiety and depressive disorders. Br. J. Psychiatry 184, 470–476. 10.1192/bjp.184.6.470 15172939

[B16] DiSantostefano J. (2009). International classification of diseases 10th revision (ICD-10). J. Nurse Practit. 5, 56–57. 10.1016/j.nurpra.2008.09.020

[B17] Dragomiretskiy K. Zosso D. (2013). Variational mode decomposition. IEEE Trans. Signal Process. 62, 531–544. 10.1109/TSP.2013.2288675

[B18] Ehman E. C. Johnson G. B. Villanueva-Meyer J. E. Cha S. Leynes A. P. Larson P. E. Z.. (2017). PET/MRI: where might it replace pet/ct? J. Magn. Reson. Imaging 46, 1247–1262. 10.1002/jmri.25711 28370695 PMC5623147

[B19] El-Dahshan E. Bassiouni M. Khare S. Tan R. Acharya U. (2024). Exhyptnet: an explainable diagnosis of hypertension using efficientnet with ppg signals. Expert Syst. Appl. 239:122388. 10.1016/j.eswa.2023.122388

[B20] Fan Y. Yu R. Li J. Zhu J. Li X. (2020). “EEG-based mild depression recognition using multi-kernel convolutional and spatial-temporal feature,” in 2020 IEEE International Conference on Bioinformatics and Biomedicine (BIBM) (IEEE: Seoul, Korea), 1777–1784. 10.1109/BIBM49941.2020.9313499

[B21] Feldmann L. Piechaczek C. Grunewald B. Pehl V. Bartling J. Frey M.. (2018). Resting frontal EEG asymmetry in adolescents with major depression: impact of disease state and comorbid anxiety disorder. Clin. Neurophysiol. 129, 2577–2585. 10.1016/j.clinph.2018.09.028 30415151

[B22] Fink P. Hansen M. Oxhoj M. (2004). The prevalence of somatoform disorders among internal medical inpatients. J. Psychosom. Res. 56, 413–418. 10.1016/S0022-3999(03)00624-X 15094025

[B23] Grieve P. Fifer W. Cousy N. Monk C. Stark R. Gingrich J.. (2019). Neonatal infant eeg bursts are altered by prenatal maternal depression and serotonin selective reuptake inhibitor use. Clin. Neurophysiol. 130, 2019–2025. 10.1016/j.clinph.2019.08.021 31539768 PMC6944195

[B24] Gureje O. Reed G. (2016). Bodily distress disorder in ICD-11: problems and prospects. World Psychiatry 15, 291–292. 10.1002/wps.20353 27717252 PMC5032513

[B25] Harris A. Orav E. Bates D. Barsky A. (2009). Somatization increases disability independent of comorbidity. J. Gen. Intern. Med. 24, 155–161. 10.1007/s11606-008-0845-0 19031038 PMC2629001

[B26] Hasanzadeh F. Mohebbi M. Rostami R. (2020). Graph theory analysis of directed functional brain networks in major depressive disorder based on EEG signal. J. Neural Eng. 17:26010. 10.1088/1741-2552/ab7613 32053813

[B27] Huijbregts K. van der Feltz-Cornelis C. van Marwijk H. de Jong F. van der Windt D. Beekman A. (2010). Negative association of concomitant physical symptoms with the course of major depressive disorder: a systematic review. J. Psychosom. Res. 68, 511–519. 10.1016/j.jpsychores.2009.11.009 20488267

[B28] Hung C. Liu C. Yang C. (2019). Persistent depressive disorder has long-term negative impacts on depression, anxiety, and somatic symptoms at 10-year followup among patients with major depressive disorder. J. Affect. Disord. 243, 255–261. 10.1016/j.jad.2018.09.068 30248637

[B29] Klooster D. Voetterl H. Baeken C. Arns M. (2023). Evaluating robustness of brain stimulation biomarkers for depression: a systematic review of mri and eeg studies. Biol. Psychiatry. 95, 553–63. 10.1016/j.biopsych.2023.09.009 37734515

[B30] Kurita G. Sjogren P. Juel K. Hojsted J. Ekholm O. (2012). The burden of chronic pain: a cross-sectional survey focussing on diseases, immigration, and opioid use. Pain 153, 2332–2338. 10.1016/j.pain.2012.07.023 22959600

[B31] Leiknes K. Finset A. Moum T. Sandanger I. (2006). Methodological issues concerning lifetime medically unexplained and medically explained symptoms of the composite international diagnostic interview: a prospective 11-year followup study. J. Psychosom. Res. 61, 169–179. 10.1016/j.jpsychores.2006.01.007 16880019

[B32] Leiknes K. Finset A. Moum T. Sandanger I. (2007). Course and predictors of medically unexplained pain symptoms in the general population. J. Psychosom. Res. 62, 119–128. 10.1016/j.jpsychores.2006.08.009 17270569

[B33] Malviya L. Mal S. (2023). CIS feature selection based dynamic ensemble selection model for human stress detection from eeg signals. Cluster Comput. 2, 1–15. 10.1007/s10586-023-04008-8

[B34] Mayou R. Kirmayer L. Simon G. Kroenke K. Sharpe M. (2005). Somatoform disorders: time for a new approach in dsm-v. Am. J. Psychiatry 162, 847–855. 10.1176/appi.ajp.162.5.847 15863783

[B35] McFarlane A. Ellis N. Barton C. Browne D. Van Hooff M. (2008). The conundrum of medically unexplained symptoms: questions to consider. Psychosomatics 49, 369–377. 10.1176/appi.psy.49.5.369 18794504

[B36] Mergl R. Seidscheck I. Allgaier A. Moller H. Hegerl U. Henkel V. (2007). Depressive, anxiety, and somatoform disorders in primary care: prevalence and recognition. Depress. Anxiety 24, 185–195. 10.1002/da.20192 16900465

[B37] Mohammadi Y. Moradi M. (2021). Prediction of depression severity scores based on functional connectivity and complexity of the eeg signal. Clin. EEG Neurosci. 52, 52–60. 10.1177/1550059420965431 33040603

[B38] Murphy M. Whitton A. E. Deccy S. Ironside M. L. Rutherford A. Beltzer M.. (2020). Abnormalities in electroencephalographic microstates are state and trait markers of major depressive disorder. Neuropsychopharmacology 45, 2030–2037. 10.1038/s41386-020-0749-1 32590838 PMC7547108

[B39] Nusslock R. Shackman A. McMenamin B. Greischar L. Davidson R. Kovacs M. (2018). Comorbid anxiety moderates the relationship between depression history and prefrontal eeg asymmetry. Psychophysiology 55:e13164. 10.1111/psyp.12953 28755454 PMC5732031

[B40] Panier L. Bruder G. Svob C. Wickramaratne P. Gameroff M. Weissman M.. (2020). Predicting depression symptoms in families at risk for depression: interrelations of posterior eeg alpha and religion/spirituality. J. Affect. Disord. 274, 969–976. 10.1016/j.jad.2020.05.084 32664041 PMC8451225

[B41] Richman J. (2011). Multivariate neighborhood sample entropy: a method for data reduction and prediction of complex data. Methods Enzymol. 487, 397–408. 10.1016/B978-0-12-381270-4.00013-5 21187232

[B42] Sharpe M. Mayou R. Walker J. (2006). Bodily symptoms: new approaches to classification. J. Psychosom. Res. 60, 35–36. 10.1016/j.jpsychores.2006.01.020 16581358

[B43] Simon G. VonKorff M. Piccinelli M. Fullerton C. Ormel J. (1999). An international study of the relation between somatic symptoms and depression. N. Engl. J. Med. 341, 1329–1335. 10.1056/NEJM199910283411801 10536124

[B44] Siuly S. Khare S. K. Kabir E. Sadiq M. T. Wang H. (2024). An efficient Parkinson's disease detection framework: leveraging time-frequency representation and alexnet convolutional neural network. Comput. Biol. Med. 174:108462. 10.1016/j.compbiomed.2024.108462 38599069

[B45] Steinbrecher N. Koerber S. Frieser D. Hiller W. (2011). The prevalence of medically unexplained symptoms in primary care. Psychosomatics 52, 263–271. 10.1016/j.psym.2011.01.007 21565598

[B46] Wang B. Kang Y. Huo D. Chen D. Song W. Zhang F. (2023). Depression signal correlation identification from different eeg channels based on cnn feature extraction. Psychiatry Res. Neuroimaging 328:111582. 10.1016/j.pscychresns.2022.111582 36565553

[B47] Wittchen H. Jacobi F. Rehm J. Gustavsson A. Svensson M. Jonsson B.. (2011). The size and burden of mental disorders and other disorders of the brain in Europe 2010. Eur. Neuropsychopharmacol. 21, 655–679. 10.1016/j.euroneuro.2011.07.018 21896369

[B48] Zhang Z. Meng Q. Jin L. Wang H. Hou H. (2024). A novel EEG-based graph convolution network for depression detection: incorporating secondary subject partitioning and attention mechanism. Expert Syst. Appl. 239:122356. 10.1016/j.eswa.2023.122356

[B49] Zhu J. Wang Y. La R. Zhan J. Niu J. Zeng S.. (2019). Multimodal mild depression recognition based on eeg-em synchronization acquisition network. IEEE Access 7, 28196–28210. 10.1109/ACCESS.2019.2901950

[B50] Zuchowicz U. Wozniak-Kwasniewska A. Szekely D. Olejarczyk E. David O. (2019). EEG phase synchronization in persons with depression subjected to transcranial magnetic stimulation. Front. Neurosci. 12:1037. 10.3389/fnins.2018.01037 30692906 PMC6340356

